# Surface Functionalization of Bamboo via Photo-Grafting Tannic Acid for Enhanced Silver Ion Loading Properties

**DOI:** 10.3390/molecules29133203

**Published:** 2024-07-05

**Authors:** Juan Xu, Lanxiang Liu, Jinju Ma, Baoshan Tang, Zhengjun Shi, Hong Zhang

**Affiliations:** 1 Institute of Highland Forest Science, Chinese Academy of Forestry, Kunming 650233, China; xujuan89@hotmail.com (J.X.);; 2 Key Laboratory for Forest Resources Conservation and Utilization in the Southwest Mountains of China, Ministry of Education, Southwest Forestry University, Kunming 650224, China

**Keywords:** bamboo, photo-grafting, tannic acid, surface functionalization, silver ion loading

## Abstract

Photo-grafting is a gentle, simple, and precise approach to incorporating specific functional molecules for the surface functionalization of substrates. In this work, ultraviolet (UV)-induced tannic acid (TA) grafting onto the surface of bamboo was proposed as a viable strategy for functionalizing bamboo. X-ray photoelectron spectroscopy (XPS) and Fourier transform infrared spectroscopy (FTIR) clearly indicated that TA was successfully introduced to the bamboo’s surface. The optimal conditions for the grafting reaction were determined to be 15 mM Methyl-2-benzoylbenzoate (BB), 30 mM TA, 20 min, and a pH = 8. Under these conditions, the amount of TA grafted onto the bamboo’s surface was measured to be 19.98 μg/cm^2^. Results from Inductively Coupled Plasma (ICP) and Energy Dispersive Spectrometer (EDS) analyses showed that the silver ion loading capacity of tannic acid-grafted bamboo was significantly improved compared to that of raw bamboo and tannic acid-impregnated bamboo. Furthermore, the presence of TA grafted on the bamboo’s surface exhibited a positive correlation with the loading of silver ions, indicating that grafted TA plays an important role in the surface functionalization of bamboo. We believe that photo-grafted TA may help generate multifunctional bamboo with diverse properties.

## 1. Introduction

Surface properties play a vital role in determining the performance of various materials, including wood, bamboo, metals, and polymers [1]. In recent decades, surface modifications have garnered significant interest as a means of altering surfaces to impart specific properties, such as wettability, surface activity, morphology, and chemical reactivity, among others. Surface modification can be achieved through both physical methods (e.g., plasma treatment, laser application, spin casting) and chemical methods (e.g., oxidation, hydrolysis, acid etching, photo-grafting) [2]. Among these methods, photo-grafting has been widely utilized due to its advantages of high efficiency, cost-effectiveness, environmental friendliness, precise controllability, and non-destructive impact on the internal structure and properties of materials [3,4].

The initiation of photopolymerization reactions heavily relies on the presence of photoinitiators (PIs), which play a crucial role in this process [5]. According to the mechanism of free radical formation, photoinitiators (PIs) can be classified into two types: Type I and Type II. Type I PIs typically cleave single molecular bonds upon irradiation to generate active free radicals, whereas Type II PIs require hydrogen abstraction reactions to generate active free radicals. Researchers select the appropriate photoinitiator based on the properties of the substrates and grafted materials to effectively carry out the photoinduced grafting reaction on the surface of the substrate [6,7,8,9,10].

Bamboo is a low-cost, rapidly growing, abundant, and renewable biomass resource. It is comparable to woody materials in terms of mechanical properties, applications, and appearance [11,12,13,14]. Moreover, bamboo and its derivatives are extensively used in construction, engineering, decoration, and everyday products. With the increasing demand for wood materials, bamboo has emerged as a viable alternative to traditional woody materials [15]. However, the sugars, starches, and proteins in bamboo render it highly susceptible to mold growth and insect infestation during processing, transportation, storage, and utilization, due to the provision of nutrients for the growth of microorganisms such as molds and stain-causing bacteria [16]. Moreover, the uneven chemical composition of bamboo’s surface hampers effective loading of antimicrobial agents onto its surface. Therefore, controlling the surface chemistry of bamboo could significantly impact the development of novel coatings and adhesives for bamboo and bamboo-based materials.

Tannic acid (TA) is a natural polyphenol commonly found in plants. Its structure consists of a central glucose molecule linked to 10 gallic acid units through ester bonds [17]. The abundance of adjacent phenolic hydroxyl groups, benzene rings, and ester moieties contributes to the high chemical reactivity of tannic acid. This allows it to interact with diverse substrate surfaces, molecules, and metal ions through non-covalent interactions, such as hydrogen bonding, hydrophobic interactions, π-π stacking, electrostatic force [18], covalent bonding, and metal–catechol coordination [19,20,21]. The versatile bonding abilities of TA make it a valuable tool for surface modification of substrates. In this study, UV-assisted photo-grafting of tannic acid (TA) was employed to functionalize the surface of bamboo, and the optimal process conditions were determined. The chemical transformations occurring on the bamboo’s surface during photo-grafting were investigated using XPS and FTIR techniques. Additionally, the silver ion loading capacity of untreated bamboo, bamboo impregnated with tannic acid solution, and bamboo photo-grafted with tannic acid was compared. Through surface functionalization, our objective is to enhance the bamboo’s capacity for carrying silver ions, thereby endowing it with exceptional antibacterial properties. This advancement holds significant implications for expanding its utilization in food packaging and water purification.

## 2. Results and Discussion

### 2.1. Surface Modification of Bamboo by TA Photo-Grafting

**Figure 1:**
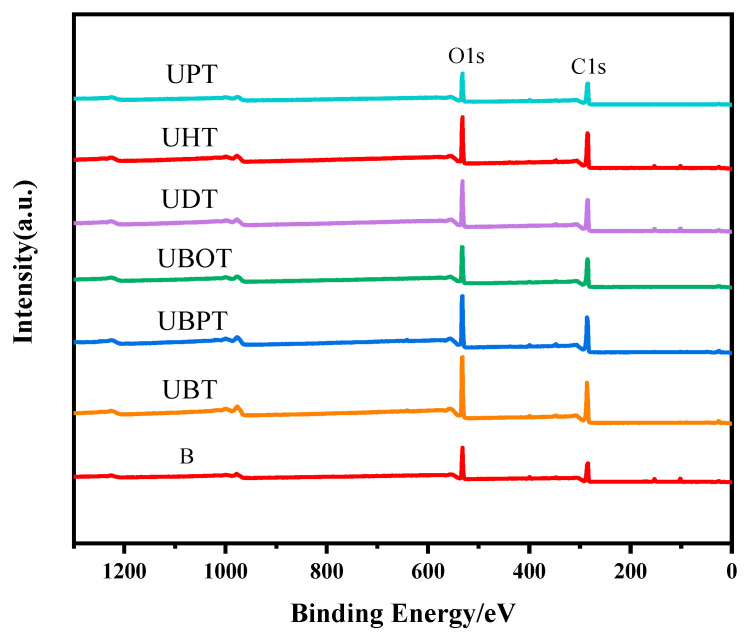
XPS spectrogram of tannic acid-grafted bamboo surface treated with various photoinitiators, such as BB (UBT), BBP (UBPT), BBO (UBOT), DPMA (UDT), HP (UHT), and PPO (UPT).

Photoinitiators play a crucial role in UV photo-grafting reactions. To elucidate the effects of different photoinitiators on the grafting of tannic acid onto bamboo surfaces, the chemical structures of bamboo treated with various photoinitiators were analyzed. The oxygen-to-carbon (O/C) ratios were determined from the XPS spectra for all samples and are presented in Figure 1 and Figure S1 and Table 1. After grafting TA, the O/C ratios for samples treated with UBT, UBPT, UBOT, UDT, UHT, and UPT were 54.18%, 49.63%, 45.03%, 42.90%, 41.46%, and 43.84%, respectively. The results indicated that the photoinitiator BB was more effective in facilitating the grafting of tannic acid onto the bamboo’s surface, while PPO had little effect on the grafting process. Consequently, BB was selected as the photoinitiator for tannic acid grafting onto bamboo surfaces.

**Figure 2:**
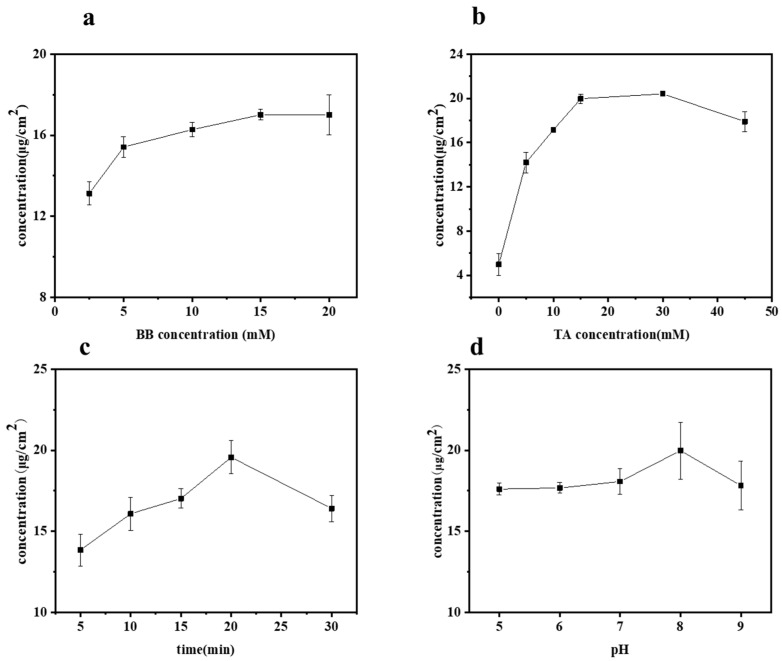
Amount of grafted TA concentration on bamboo surface as a function of (**a**) BB concentration, (**b**) TA concentration, (**c**) UV irradiation time, and (**d**) pH value.

The influences of BB concentration, TA concentration, UV irradiation time, and pH value on the grafting of TA onto bamboo surfaces were studied by measuring the TA content on various samples, with the results presented in Figure 2a–d. Specifically, the effect of BB concentration on the grafting of TA was investigated at different concentrations: 2.5, 5, 10, 15, and 20 mM, as shown in Figure 2a. During these tests, all other parameters, such as TA concentration, UV irradiation time, and pH value, were held constant at 10 mM, 20 min, and pH 8, respectively. As expected, the content of TA on the bamboo’s surface increased with the increase in BB concentration. The TA concentration ranged from 13.13 to 17.01 μg/cm^2^ when the BB concentration varied from 2.5 to 15 mM. However, beyond a BB concentration of 15 mM, the TA content gradually stabilized. Considering both the cost and the effective concentration of grafted TA, the optimal BB concentration was selected as 15 mM.

The effect of varying tannic acid (TA) concentrations (0, 5, 10, 15, 30, and 45 mM) on the grafted TA concentration was studied, as shown in Figure 2b. This was performed at a constant BB concentration (15 mM), UV irradiation time (20 min), and pH value (8). When the TA concentration was below 30 mM, the efficiency of TA grafting onto the bamboo’s surface increased with the solution concentration. However, beyond a 30 mM TA concentration, the grafted TA concentration dropped from 20.42 to 17.91 μg/cm^2^. Notably, lower concentrations of TA resulted in a higher grafting content compared to higher concentrations. This phenomenon occurs because tannic acid exists as a molecular dispersion at low concentrations, whereas at higher concentrations, it forms colloids, resulting in a molecular aggregation state [22]. The aggregation state is not conducive to the grafting reaction of tannic acid. Therefore, we selected a TA concentration of 30 mM for this experiment.

Figure 2c illustrates the concentration of grafted TA on the bamboo’s surface as a function of irradiation time. As expected, at a constant BB concentration (15 mM), TA concentration (30 mM), and pH value (8), the trend generally increased initially and then decreased with prolonged irradiation time (5, 10, 15, 20, 30 min). It was observed that the grafted TA concentration increased from 13.85 to 19.57 μg/cm^2^ as the irradiation time extended from 5 to 20 min. The content of tannic acid grafted onto the bamboo’s surface peaked at an irradiation time of 20 min. However, extending the irradiation to 30 min resulted in a decrease in the grafted TA concentration to 16.41 μg/cm^2^. This decrease can be attributed to the inherent instability of tannic acid, which can decompose under prolonged exposure to UV light. After careful consideration, an irradiation time of 20 min was selected for this experiment.

The effects of different pH values (5, 6, 7, 8, 9) were studied under fixed conditions of BB concentration (15 mM), TA concentration (30 mM), and UV irradiation time (20 min), as shown in Figure 2d. pH value played a dominant role; the grafted TA concentration increased slightly from 17.61 to 18.07 μg/cm^2^ as the pH rose to below 8. However, the grafted TA concentration peaked at 19.98 μg/cm^2^ at a pH of 8, indicating that TA is more readily grafted onto the bamboo surface at this higher pH. This phenomenon could be attributed to the fact that under alkaline conditions, TA undergoes oxidation of its adjacent phenolic hydroxyl groups to form quinones, which facilitates grafting [23,24], and subsequently, generates quinone radicals. In the case of photoinitiation, these free radicals readily bond with the bamboo’s surface.

### 2.2. Chemical Changes Due to UV Photo-Grafting Process

The elemental composition of a solid surface can be qualitatively or semi-quantitatively analyzed using X-ray photoelectron spectroscopy (XPS). Bamboo consists of cellulose, hemicellulose, lignin, and extractives, with carbon, oxygen, and hydrogen being the primary elements in bamboo, BB, and TA [25]. Therefore, changes in peak intensities and elemental compositions can provide insights into the functional group structure and chemical properties of the bamboo’s surface.

**Figure 3:**
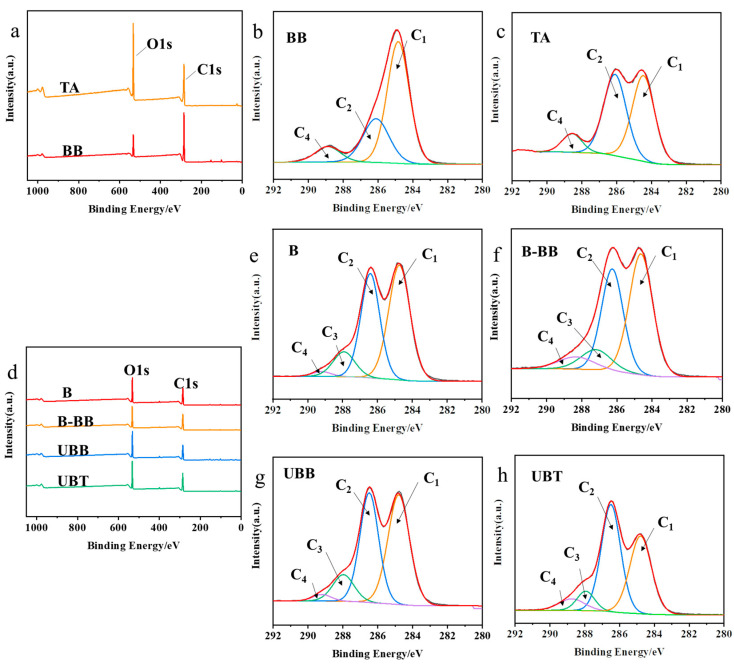
(**a**) XPS spectrogram of BB and TA; C1’s spectra of carbon peak component of reagents (**b**) BB, (**c**) TA; (**d**) XPS spectrogram of B, B-BB, UBB, UBT; C1’s spectra of carbon peak component during the grafting process of tannic acid onto the surface of bamboo (**e**) B, (**f**) B-BB, (**g**) UBB, (**h**) UBT.

The XPS spectra of reagent BB, TA, and bamboo samples (B, B-BB, UBB, and UBT) are presented in Figure 3. In order to gain a deeper understanding of the functional groups present, the C1s spectra were decomposed into three or four peaks that corresponded to C1 (C-C or/and C-H), C2 (C-O), C3 (O-C-O), and C4 (O-C=O) [26]. The peak of C-O might correspond to the presence of cellulose in bamboo, while the C=O peak was attributed to lignin on bamboo (Figure 3e) [27]. The fraction area of each peak’s components is summarized in Table 2. Compared with B, the carbon signals of UBB significantly increased at C2, C3, and C4, and were significantly reduced at C1, indicating that bamboo was successfully grafted with BB. Similarly, the C1 contribution decreased from 48.56% to 38.40%, the intensities of the C2 and C4 peaks in UBT increased from 38.99% to 46.96% and from 1.75% to 6.85% (Figure 3h), respectively, and compared with UBB, the C2 and C4 proportions of UBT increased from 40.18% to 46.96%, and from 2.38 to 6.85. The O/C ratio increased from 43.58% to 51.51%, both of which could be associated with the hydroxyl and ester groups present in the TA grafted on the bamboo’s surface [28].

**Figure 4:**
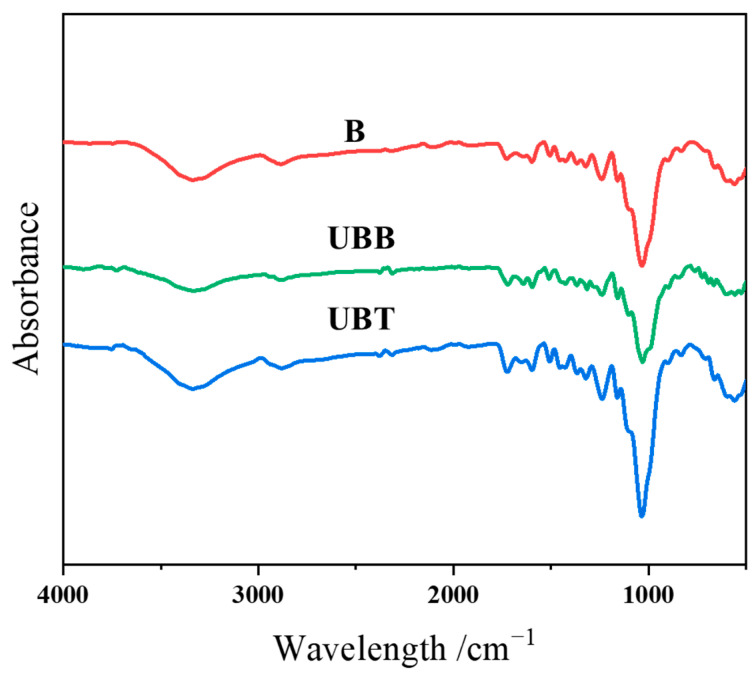
FTIR spectrogram of B, UBB, UBT.

The grafting of TA on the bamboo’s surface was further determined by ATR-FTIR; the spectra of B, UBB, and UBT are shown in Figure 4. The characteristic peaks near 3340, 1725, and 1160 cm^−1^ correspond to the O-H, C=O, and C-O-C functional groups, respectively [12,29]. The peaks at 1600 and 1506 cm^−1^ were attributed to the aromatic rings, and the C-O peaks at 1240 and 1030. Moreover, the peaks at 2925, 2881, 1375, and 900 cm^−1^ were the vibrations of C-H bonds [30]. Since the peak at 898 cm^−1^ for β-glucoside linkage of cellulose in bamboo is prominent and assumed to be unaffected during chemical treatment, this peak was chosen as an internal standard [31,32]. The ATR-FTIR spectra were normalized according to the peak intensity of the internal standard peak, and the corresponding absorbance intensity ratios (*I*_v_/*I*_898_) are summarized in Table 3. After TA treatment, the absorbance intensity ratio for -OH vibration increased from 6.67 to 7.67, and the peak intensity ratios corresponding to C=O (1725 cm^−1^), asymmetrical C-O-C (1160 cm^−1^), and C-O stretching (1240 and 1030 cm^−1^) were observed to be increased. Furthermore, the intensity ratio of the peak at 1600 cm^−1^ for the aromatic carbon–carbon double bonds was found to be changed from 2.33 to 3.27 after grafting TA. There was an observed increase in the absorbance area ratios for all of these peaks for asymmetric stretching after immobilization with TA onto the bamboo’s surface.

### 2.3. Analysis of Silver Ion Loading Capacity on Modified Bamboo Surface

**Figure 5:**
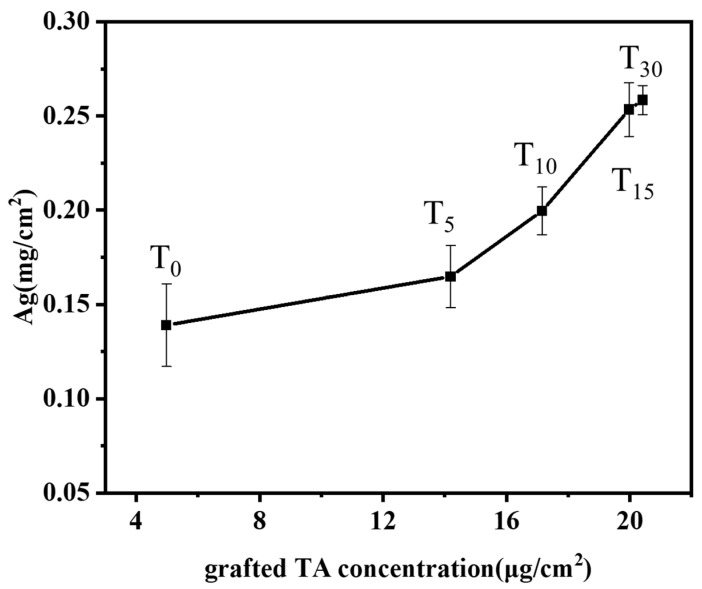
Effects of grafting TA concentration of bamboo surface on the loading capacity of silver ions.

TA-grafted bamboo samples were prepared with various TA solution concentrations (0, 5, 10, 15, 30 mM, labeled T_0_, T_5_, T_10_, T_15_, T_30_) at the optimum conditions of BB concentration (15 mM), irradiation time (20 min), and pH 8. The influence of varying the grafted tannic acid concentration of the bamboo’s surface on the silver ion loading capacity is shown in Figure 5. The results depicted demonstrate that the loading of silver ions on the surface of bamboo increases with the increasing concentration of grafted tannic acid. Even in the absence of added tannic acid (T_0_), the bamboo’s surface exhibits a certain affinity for silver ions with a concentration of 0.14 mg/cm^2^. This affinity can be attributed to the inherent lignin present on the bamboo’s surface, whose phenolic hydroxyl groups are capable of binding with silver ions. Moreover, since the phenolic hydroxyl groups in lignin can react with the Folin–Ciocalteu reagent to produce a color, the presence of phenolic hydroxyl activity can be detected even in the absence of tannic acid. Consequently, the binding of silver ions to the phenolic hydroxyl groups of lignin is observable in the control group as well. Upon increasing the TA concentration to 30 mM, the silver ion content significantly rises to 0.26 mg/cm^2^, indicating a roughly doubled increase. Correspondingly, under these conditions, the grafting density of tannic acid onto the bamboo’s surface was also found to be at its peak (Figure 2). In conclusion, the presence of TA grafted onto the bamboo’s surface exhibits a positive correlation with the loading of silver ions.

**Figure 6:**
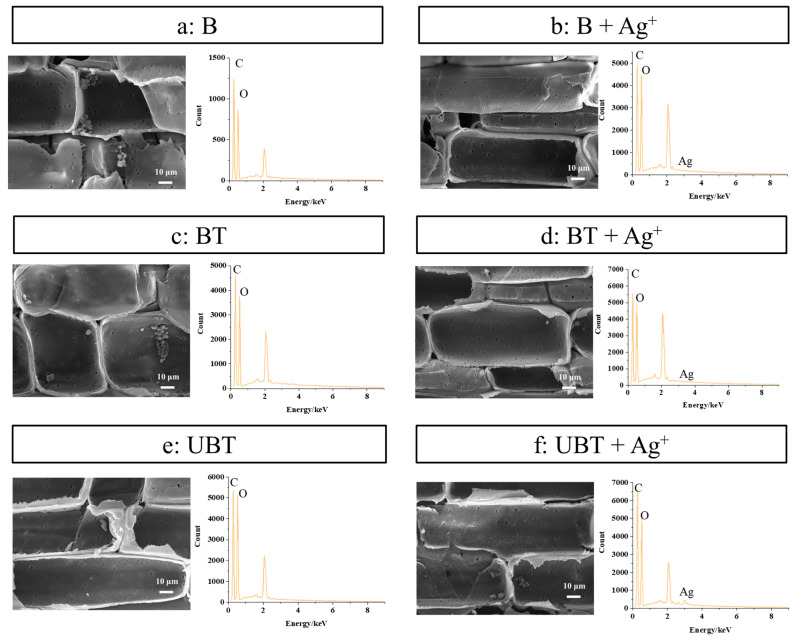
SEM and EDS images of the bamboo samples (**a**) B, (**b**) B loaded with silver ions, (**c**) BT, (**d**) BT loaded with silver ions, (**e**) UBT, (**f**) UBT loaded with silver ions.

SEM micrographs and EDS analyses of untreated bamboo (B), TA-impregnated bamboo (BT), and TA-photo-grafted bamboo (UBT) before and after loading of silver ions were observed and are shown in Figure 6. As can be seen from Figure 6, the bamboo treated with TA and silver ions exhibits a relatively smooth surface compared to the untreated bamboo (Figure 6a), while still displaying visible pits and retaining starch particles within the cell wall. This observation indicates that the surface modification method does not compromise the surface morphology of bamboo, thereby preserving its distinctive micromorphology. Furthermore, the content of silver, which did not exist in EDS images of B, BT, and UBT (Figure 6a,c,e, and Table 4), were 0.03%, 0.18%, and 0.38% in EDS graphs of B, BT, and UBT after loading silver ions (Figure 6b,d,f), respectively. Whether TA was introduced through immersion and adsorption or grafted onto the bamboo’s surface using UV, the presence of tannic acid on the bamboo’s surface enhances the loading capacity of silver ions. However, bamboo grafted with photoinitiated TA exhibits a significantly higher loading capacity, approximately 1 times that of BT. This enhancement is likely due to the differential interaction between tannic acid and the bamboo’s surface. For the BT samples, the main interaction between tannic acid and the bamboo’s surface is hydrogen bonding [33,34], which is subsequently compromised during the washing process, resulting in the removal of a significant portion of the tannic acid. Conversely, the UBT samples utilize a photoinitiated grafting technique, which effectively anchors tannic acid molecules to the bamboo’s surface through the formation of stable covalent bonds. These covalent bonds exhibited exceptional stability during the washing phase, leading to a higher retention of tannic acid on the bamboo’s surface. As evidenced by the data presented in Figure 5, there exists a direct correlation between the silver ion loading capacity of the bamboo’s surface and the concentration of tannic acid, thereby accounting for the superior silver ion loading capacity observed in the UBT samples. The result showed that the application of UV irradiation on the bamboo’s surface with grafted TA facilitated a more favorable loading of silver ions.

## 3. Materials and Methods

### 3.1. Materials and Chemicals

Three-year-old bamboo (*Dendrocalamus giganteus*) from Lincang city, Yunnan Province, China, was collected as the raw material in this study (no mildew, no wilt, no trauma, no moth, etc.). In order to ensure the quality of bamboo pieces, the epidermis and endodermis of bamboo were sequentially removed, then cut with an average length, width, and thickness of 10 mm × 10 mm × 2 mm. The bamboo samples (B) were dried at 60 °C for 24 h.

Methyl-2-benzoylbenzoate (BB), 4-benzoylbiphenyl (BBP), benzoyl peroxide (BP), benzoin dimethyl ether (DMPA), 1-hydroxycyclohexyl phenyl ketone (HP), phenylbis (2,4,6-trimethylbenzoyl) phosphine oxide (PPO), Folin–Ciocalteu, and silver nitrate were purchased from Macklin (Shanghai, China). Tannic acid (TA) was provided by Aladdin Industrial Corporation (Shanghai, China). All remaining solvents and chemicals were of analytical grade.

### 3.2. Surface Modification of Bamboo by TA Photo-Grafting

The bamboo pieces were placed in a Soxhlet extractor containing acetone and treated at 60 °C for several hours until the solution turned transparent. Subsequently, the bamboo block was removed and placed in a fume hood to allow the residual solvent to evaporate, followed by drying and setting it aside.

**Figure 7:**
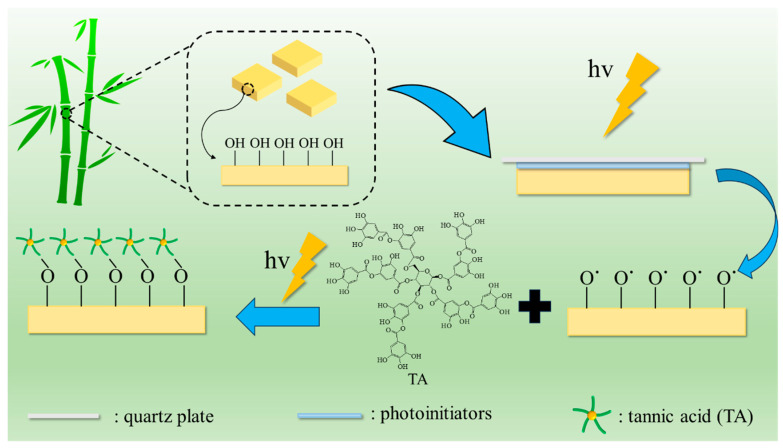
Schematic illustration of the TA photo-grafting on bamboo’s surface.

Surface modification of bamboo by TA photo-grafting was performed as shown in Figure 7. In detail, select some pre-treated bamboo pieces and place them in petri dishes. Add 100 μL of an appropriate concentration of photoinitiator acetone solution and cover with a quartz glass sheet (B-BB), and then expose to ultraviolet light for 3 min (UBB). Next, add 100 μL of tannic acid solution with an appropriate concentration and pH (pH was adjusted with 10% NaOH solution), then cover again with a quartz glass sheet. After exposing to ultraviolet light (CEL-HXF300-T3, China education Au-light, Beijing, China) for a few more minutes, they were removed. The bamboo samples were subsequently rinsed three times with acetone and underwent ultrasonic washing in deionized water until no free tannic acid was produced. The treated bamboo samples were placed in an oven at 60 °C for 24 h. Subsequently, they were transferred to a dryer for cooling before being utilized further.

The effects of the kind of photoinitiator (BB, BBP, BPO, DMPA, HP, and PPO, the corresponding samples are labeled UBT, UBPT, UBOT, UDT, UHT, and UPT), BB concentration (2.5, 5, 10, 15, 20 mM), TA concentration (5, 10, 15, 30, 45 mM), UV irradiation time (5, 10, 15, 20, 30 min), and pH value (5, 6, 7, 8, 9) on TA grafted on the bamboo’s surface were also tested.

### 3.3. Folin-Ciocalteu Quantification of TA on Samples’ Surface

The Folin–Ciocalteu method was used to determine the total phenol content on the surface of functionalized bamboo samples [35,36]. In brief, functionalized samples were immersed in a mixture of 8 mL distilled water and 0.5 mL Folin–Ciocalteu for a duration of 10 min; the addition of 1.5 mL sodium carbonate solution (Na_2_CO_3_, 7.5% *w*/*v*) followed. Subsequently, the samples were incubated at room temperature for a period of 2 h, after which absorbance was measured at 765 nm using a multilabel counter (Microplate Reader, Thermo Scientific, Waltham, MA, USA). Using gallic acid (GA) as a reference, a straight calibration line was produced, and the results were expressed as μg GA equivalents per cm^2^ of the bamboo samples.

### 3.4. Analysis of Silver Ion Loading Capacity on Modified Bamboo Surface

TA-impregnated bamboo samples’ (BT) preparation: The selected pre-treated bamboo slices were placed in petri dishes, with three pieces per dish. To each petri dish was added 3 mL of a 30 mM and pH 8 tannic acid aqueous solution. Subsequently, the petri dishes containing the samples were placed at room temperature (25 °C) for 12 h in the dark. After that, the samples were removed and thoroughly washed with deionized water until no producing free tannic acid. Finally, the obtained samples were oven-dried at 60 °C for 24 h, and then removed and cooled in a dryer for further use.

The loading of silver ions: 100 μL of a 10 g/L AgNO_3_ solution was dropped onto each sample’s (B, BT, and UBT) surface; they were stood at room temperature for 30 min. For samples B and BT, AgNO_3_ could be dropped on either side. However, for sample UBT, AgNO_3_ should be dropped on the side that still contains tannic acid. Subsequently, the samples were rinsed several times with deionized water. The resulting liquid was collected and its volume adjusted to 100 mL, and then stored at 4 °C until use. Inductively coupled plasma mass spectroscopy (ICP-MS, Thermo Scientific, Waltham, MA, USA) was used to measure the content of silver ions in the collected solution. The results were expressed as mg Ag per cm^2^ of the bamboo samples.

### 3.5. Characterizations

The chemical structures of the samples’ surface were characterized by X-ray photoelectron spectra (XPS, K-Alpha, Thermo Scientific, Waltham, MA, USA) and Fourier transform infrared (FTIR, Tenson 27, Bruker, Germany). XPS data were collected using a monochromatic Al–Kα X-ray source (1486.6 eV, 12 kV). FTIR spectral data were acquired in the ATR scanning mode with a resolution of 4 cm^−1^ and 32 scans, covering a range from 4000 to 400 cm^−1^. The surface morphology and elemental mapping were analyzed using scanning electron microscopy (SEM, Mira, Tescan, EU, Brno, Czechia) coupled with X-ray energy dispersive spectrometry (EDS), after being gold coated, and the accelerated voltages for surface morphology and energy spectrum mapping were 3 kV and 15 kV, respectively.

## 4. Conclusions

In this study, we introduced a straightforward and efficient strategy for modifying the surface of bamboo. The grafting of tannic acid (TA) onto the bamboo’s surface was successfully achieved through the catalysis of a photoinitiator. Analytical results from both X-ray photoelectron spectroscopy (XPS) and Fourier Transform Infrared Spectroscopy (FTIR) confirmed the presence of characteristic peaks corresponding to the atoms and functional groups of TA, allowing us to select the optimal conditions for the surface-grafting process. Compared to untreated bamboo and TA-impregnated bamboo, the photoinitiated grafting method showed an enhanced ability to support silver ions on the bamboo’s surface. This indicates that grafting TA onto the bamboo’s surface is more conducive to loading silver ions. Furthermore, this study demonstrated that functionalized TA not only supports the loading of silver ions but also facilitates secondary modifications and enhances the surface properties of bamboo.

## Figures and Tables

**Table 1:** Relative amount of atoms and O/C ratio of tannic acid-grafted bamboo surface treated with various photoinitiators.

Photoinitiators	TA-Grafted Bamboo Samples	C (%)	O (%)	O/C (%)
/	B	68.30	31.70	46.41
BB	UBT	64.86	35.14	54.18
BBP	UBPT	67.09	32.91	49.05
BBO	UBOT	68.95	31.05	45.03
DMPA	UDT	69.98	30.02	42.90
HP	UHT	70.69	29.31	41.46
PPO	UPT	69.52	30.48	43.84

**Table 2:** C1’s division peak percentage content and O/C ratio of BB, TA, and the samples produced during the process of tannic acid-grafted bamboo surface.

Samples	Carbon Components				O/C (%)
	C1/%	C2/%	C3/%	C4/%	
BB	63.72	27.12	/	9.17	21.77
TA	46.50	44.54	/	8.96	56.57
B	48.56	38.99	10.69	1.75	43.58
B-BB	46.51	36.30	10.04	7.15	42.63
UBB	46.38	40.18	11.06	2.38	49.60
UBT	38.40	46.96	7.79	6.85	51.51

**Table 3:** ATR-FTIR spectral data and absorbance intensity ratios of B and UBT.

Position of Bands (cm^−1^)	Possible Assignments	B (*I*_v_/*I*_898_)	UBT (*I*_v_/*I*_898_)
3340	-OH stretching	6.67	7.67
2920	-CH stretching in methyl and methylene	2.07	2.33
1725	Non-conjugated C=O stretching	2.27	4.33
1600	Aromatic -C=C- stretching	2.33	3.27
1506	Aromatic -C=C- stretching	2.33	3.20
1240	C–O stretching	5.40	7.07
1160	C–O–C stretching	3.13	3.87
1030	Aromatic C–H, symmetrical C–O stretching	16.13	24.47

**Table 4:** Relative amount of atoms before and after loading silver ions in samples B, BT, and UBT determined by EDS.

Samples	C (%)	O (%)	Ag (%)
B	63.85	36.15	/
B + Ag^+^	60.21	39.76	0.03
BT	61.58	38.39	/
BT + Ag^+^	60.42	39.41	0.18
UBT	60.21	39.76	/
UBT + Ag^+^	62.23	37.38	0.38

## Data Availability

Data are contained within the article and Supplementary Materials.

## Supplementary Materials

The following supporting information can be downloaded at: https://www.mdpi.com/article/10.3390/molecules29133203/s1, Figure S1: XPS spectra with C1s region for B, UBT, UBPT, UBOT, UDT, UHT, UPT.
