# Supplementary material for: Surface Functionalization of Bamboo via Photo-Grafting Tannic Acid for Enhanced Silver Ion Loading Properties
Source: Molecules. 2024 Jul 5;29(13):3203. doi: 10.3390/molecules29133203 (PMC11243365; doi:10.3390/molecules29133203)
Supplement: Supplementary file 1 [file molecules-29-03203-s001.zip › molecules-3070244-supplementary.pdf]

Surface Functionalization of Bamboo via Photo-Grafting Tannic Acid for Enhanced Silver Ion Loading Properties

Juan Xu [1,2], Lanxiang Liu [1], Jinju Ma [1], Baoshan Tang [1], Zhengjun Shi [2,*] and Hong Zhang [1,*]

[1] Institute of Highland Forest Science, Chinese Academy of Forestry, Kunming 650233, China; xujuan89@hotmail.com (J.X.)

[2] Key Laboratory for Forest Resources Conservation and Utilization in the Southwest Mountains of China, Ministry of Education, Southwest Forestry University, Kunming 650224, China

* Correspondence: shizhengjun1979@swfu.edu.cn (Z.S.); kmzhh@hotmail.com (H.Z.)

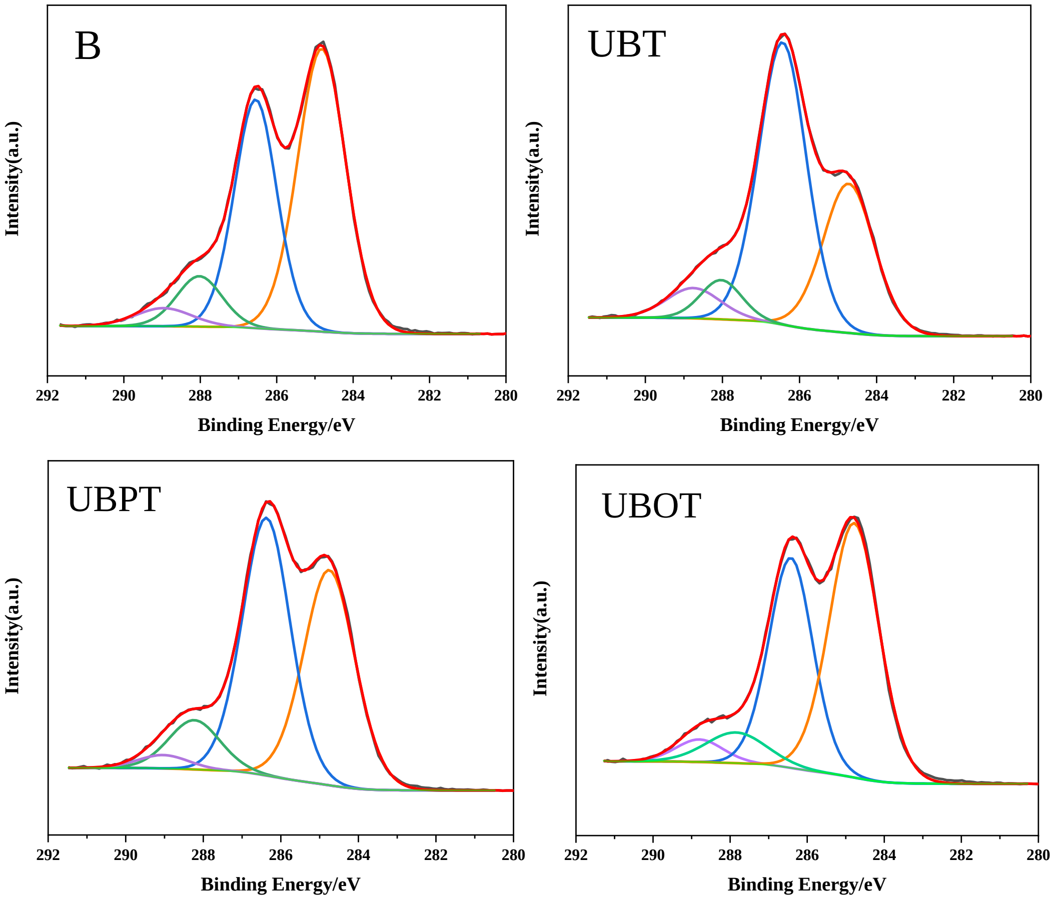

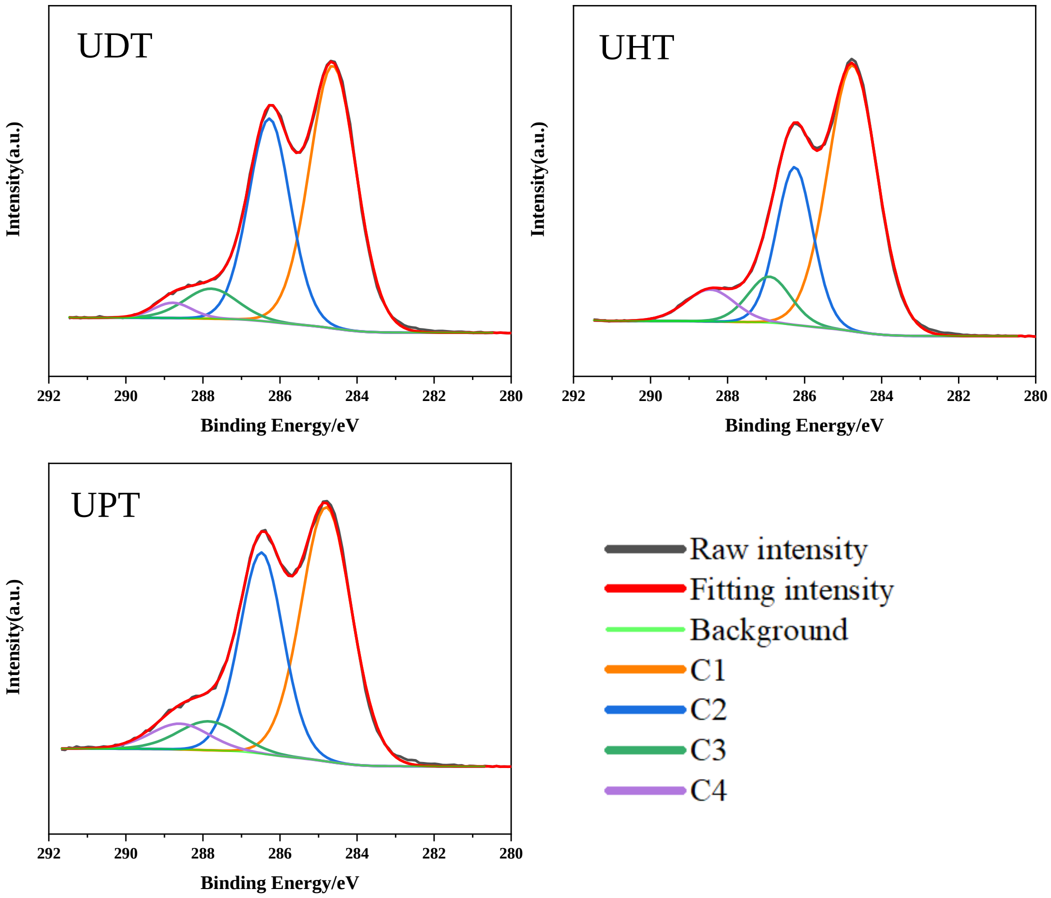

Figure S1. XPS spectra with C1s region for B, UBT, UBPT, UBOT, UDT, UHT, UPT.

.
